# Impact of two antimicrobial susceptibility reporting strategies on treatment of bacteremia caused by low-risk AmpC inducible organisms

**DOI:** 10.1017/ash.2025.49

**Published:** 2025-02-28

**Authors:** Jai Mashru, Marion Elligsen, Nick Daneman, Marie-Félixe Granger, Robert A. Kozak, Xena X. Li, Jerome A. Leis, Philip W. Lam

**Affiliations:** 1 Department of Medicine, University of Toronto, Toronto, Canada; 2 Department of Pharmacy, Sunnybrook Health Sciences Centre, Toronto, Canada; 3 Division of Infectious Diseases, Sunnybrook Health Sciences Centre, Toronto, Canada; 4 Department of Microbiology, Sunnybrook Health Sciences Centre, Toronto, Canada; 5 Shared Hospital Laboratory, Toronto, Canada

## Abstract

In this retrospective study examining the treatment of low-risk AmpC-producing Enterobacterales bacteremia during two periods with different microbiology reporting strategies, reporting of ceftriaxone susceptibility was associated with a statistically significant decrease in carbapenem use as definitive therapy compared to when susceptibility was suppressed (21 vs 50%, *p* < 0.0001).

## Introduction

AmpC beta-lactamase-producing Enterobacterales (AmpC-E) are capable of developing resistance through production of the AmpC beta-lactamase enzyme.^1^ Amp-C-E organisms have historically included *Serratia* species, *Providencia* species*, Proteus vulgaris, Citrobacter freundii complex, Enterobacter cloacae complex, Klebsiella aerogenes*, and *Morganella morganii.* ^1,2^ These organisms may appear susceptible to third-generation cephalosporins initially but can develop resistance following beta-lactam exposure.

A subset of AmpC-E (*Serratia*, *Providencia* and *Morganella* species), have lower rates of AmpC expression and inducibility. A 2018 *in vitro* study estimates there is a very low rate of selection for AmpC-derepressed mutants during beta-lactam therapy whilst older *in vivo* studies have suggested less than 5% of these organisms confer inducible resistance.^3,4^ Recently, the Infectious Disease Society of America (IDSA) suggested that these “low-risk” AmpC-E can be treated according to susceptibility results.^5^

This updated guideline introduces an opportunity for antimicrobial stewardship whereby low-risk AmpC-E can be treated with narrower spectrum beta-lactams, sparing use of carbapenems. The objective of this study was to characterize the treatment of bacteremia caused by low-risk AmpC-E at our institution during two time periods with different antimicrobial susceptibility reporting strategies.

## Methods

We conducted a retrospective cohort study examining adult inpatients with positive blood cultures growing *Serratia*, *Morganella*, and *Providencia* species at Sunnybrook Health Sciences Centre (a 718-acute-bed tertiary academic center) between January 1, 2012, and December 31, 2021.

During the study period, two different standard operating procedures were utilized by the microbiology laboratory to report antibiotic susceptibilities for AmpC-E. These changes pre-dated the updated IDSA treatment guidelines on AmpC-E and were related to changes in laboratory management. From January 1, 2012 to August 31, 2019 (ceftriaxone reporting period), ceftriaxone susceptibility results were reported for all AmpC-E along with the comment: “*Resistance to all penicillins, beta lactamase inhibitors and cephalosporins may develop during therapy with these agents*”. Carbapenem susceptibility results were not routinely reported. From September 1 2019 to December 31, 2021 (ceftriaxone suppression period), ceftriaxone susceptibility results were hidden, and ertapenem susceptibility was routinely reported for all AmpC-E. The report included the following comment: *“This organism is generally considered resistant to all penicillins, cephalosporins and beta lactam/lactamase inhibitor combinations”*. Ciprofloxacin, trimethoprim-sulfamethoxazole, and aminoglycoside susceptibility results were routinely reported during both time periods. No other interventions affecting antibiotic prescribing for AmpC-E were introduced during the study period.

Patient and microbiology data were extracted from an antimicrobial stewardship database^6^, and a chart review was conducted to collect initial and definitive antibiotic treatment regimens, all-cause 30-day mortality, and recurrent bacteremia within 30 days of index bacteremia. The initial antibiotic regimen was defined as the first antibiotic directed against gram-negative organisms prescribed after blood cultures were drawn, whereas the definitive antibiotic regimen was defined as the antibiotic prescribed to complete the course of treatment after susceptibility results were available. Recurrent bacteremia was defined as a repeat episode of bacteremia occurring in a patient with an infection-free interval between the two episodes.

The primary outcome was the proportion of bacteremia episodes treated with carbapenems as definitive therapy. Secondary outcome measures included the proportion of bacteremia episodes treated with other antibiotic classes, duration of therapy, all-cause mortality, and recurrent bacteremia at 30 days. Only antimicrobials with activity against low-risk AmpC-E were included in treatment duration calculations. Outpatient oral stepdown contributed to treatment duration calculations. Descriptive analyses were used to characterize the cohort during the two time periods. Median with interquartile ranges and proportions were used for continuous and categorical variables, respectively. Comparisons between the two time periods were carried out using the Chi-square test. Approval for this study was obtained from the institutional research ethics board.

## Results

There were 244 unique blood cultures growing *Serratia*, *Providencia,* and *Morganella* species identified during the study period. *Serratia marcescens* accounted for 68% of positive blood cultures. Almost all isolates (235 of 244, 96%) were susceptible to ceftriaxone and piperacillin-tazobactam. Ciprofloxacin resistance was low in both periods (8% and 4%). The median age of the patients who developed bacteremia was 71 years and 69% of patients were male. Patient and clinical characteristics are summarized in Table 1.

**Table 1.** Characteristics of patients with low-risk AmpC beta-lactamase-producing Enterobacterales bacteremia during two time periods

Variable	Ceftriaxone reporting period (%) *n = 196*	Ceftriaxone suppression period (%) *n = 48*
Age, median (IQR)	71 (59, 79)	70 (59, 78)
Male	130 (66)	38 (79)
Organisms		
*Serratia marcescens*	130 (66)	36 (75)
*Morganella morganii*	47 (24)	9 (18)
*Providencia stuartii*	9 (5)	1 (2)
*Providencia rettgeri*	6 (3)	1 (2)
*Serratia liquefaciens*	4 (2)	1 (2)
Site where positive blood culture drawn		
Peripheral venipuncture	171 (87)	32 (67)
Central venous catheter	10 (5)	5 (10)
Arterial catheter	6 (3)	9 (19)
Peripherally inserted central catheter	3 (2)	2 (4)
Hemodialysis catheter	6 (3)	0

The most common initial agents prescribed were piperacillin-tazobactam (105/244, 43%), ceftriaxone (52/244, 21%) and meropenem (32/244, 13%), whereas the most common definitive agents prescribed were ciprofloxacin (122/244, 50%), meropenem (26/244, 15%), and ertapenem (30/244, 12%). The distribution of antimicrobial agents prescribed initially and definitively during the two time periods is summarized in Table 2. Carbapenem use as definitive therapy was significantly lower in the ceftriaxone reporting period (42/196, 21%) compared to the ceftriaxone suppression period (24/48, 50%) (p-value< 0.0001). The median duration of therapy was 11 days (interquartile range [IQR] 8, 16), and was similar between the two time periods (ceftriaxone reporting period: 11 [IQR 7, 16], ceftriaxone suppression period: 11.5 [IQR 8, 17], *P* = 0.38).

**Table 2.** Initial and definitive antibiotics used during two times periods for treatment of low-risk AmpC beta-lactamase-producing Enterobacterales bacteremia

Antibiotic Class	Ceftriaxone reporting period (%) *n = 196*	Ceftriaxone suppression period (%) *n = 48*	p-value
Initial Antibiotic Therapy			
Carbapenems	21 (11)	17 (35)	<0.0001
Fluoroquinolones	17 (9)	3 (6)	0.58
Aminoglycosides	12 (6)	2 (4)	0.60
Extended-spectrum beta-lactam/beta-lactamase inhibitors and cephalosporins*	134 (68)	26 (54)	0.06
Other^†^	12 (6)	0	0.08
Definitive Antibiotic Therapy			
Carbapenems	42 (21)	24 (50)	<0.0001
Fluoroquinolones	105 (54)	19 (40)	0.08
Aminoglycosides	2 (1)	1 (2)	0.55
Extended spectrum beta-lactam/beta-lactamase inhibitors and cephalosporins*	24 (12)	4 (8)	0.45
Trimethoprim-Sulfamethoxazole	7 (4)	0	0.18
Other^†^	6 (3)	0	0.22

* Piperacillin-Tazobactam, ceftriaxone, and ceftazidime.

† Included: cefazolin and amoxicillin-clavulanate.

Of the patients where follow-up data was available (n=233, 96%), recurrent bacteremia occurred in 12 (5%) cases within 30 days of index infection. This was similar between both time periods (ceftriaxone reporting period: 11/188 [6%], ceftriaxone suppression period: 1/45 [2%], *P* = 0.32) (Table 1). In all cases of recurrent bacteremia (12/12), repeat susceptibility testing showed the same susceptibility pattern as the initial bacteremia. All-cause mortality at 30 days was also similar between both periods (ceftriaxone reporting period: 35/188 [19%], ceftriaxone suppression period: 12/45 [27%], *P* = 0.35).

## Discussion

In this 10-year retrospective study where two different antimicrobial susceptibility reporting strategies were used, reporting of ceftriaxone susceptibility and suppression of carbapenem susceptibility was associated with a significant reduction in carbapenem use.

The ceftriaxone reporting period of the study is aligned with the updated IDSA guidelines and thus provides an estimate of the impact of this change on antimicrobial stewardship.^5^ The high susceptibility rates identified from this cohort are consistent with published literature,^5^ with 96% of isolates susceptible to ceftriaxone and piperacillin-tazobactam, and no evidence of phenotypic resistance in the cases of recurrent bacteremia.

Our study adds to the emerging evidence that modifications in microbiology reporting cascades can profoundly influence the selection of antimicrobial therapy^7,8^. Antibiotic susceptibility reporting affirmed use of narrow-spectrum beta lactam therapy by prescribers. Conversely suppressing these results had the reverse effect. The role of the comment informing clinicians of the potential risk of resistance developing while on therapy may also affect antibiotic prescribing practices. Our findings also suggest that microbiology reporting can affect empiric antibiotic decisions in patients known to harbor AmpC-E. We are currently completing a prospective study examining the impact of an updated microbiology reporting cascade targeting low-risk AmpC-E during a subsequent time period.

There are several limitations associated with this study. First, this is a single-center retrospective study, which may limit generalizability to other centers. Second, our study only accounted for bloodstream infections and therefore underestimated the overall antimicrobial use for other infections caused by low-risk AmpC-E. Third, the small sample size limited our ability to detect differences between the two groups. Finally, due to limitations in chart abstraction, we were unable to collect additional variables that may have influenced the choice of definitive antibiotic therapy.

Aligning antibiotic susceptibility reporting with the latest IDSA recommendations on low-risk AmpC-E has the potential to reduce carbapenem use. Further studies are needed to determine the impact of these changes in microbiology reporting on clinical outcomes and antimicrobial utilization.
